# Combined Serum Expression of lncRNA CBR3‐AS1 and miR‐3163: Prognostic Significance and Underlying Mechanism in Severe Pneumonia

**DOI:** 10.1111/crj.70206

**Published:** 2026-07-07

**Authors:** Chao Liu, Meie Li, Junfa Yang, Pan Long

**Affiliations:** ^1^ Department of Emergency Medicine The Affiliated Hospital of Southwest Medical University Sichuan China; ^2^ Department of Respiratory and Critical Care Medicine People's Hospital of Fengjie Fengjie China; ^3^ Department of Respiratory and Critical Care Medicine the Affiliated Taizhou People's Hospital of Nanjing Medical University, Taizhou School of Clinical Medicine, Nanjing Medical University Taizhou China; ^4^ Department of ICU Shanghai Songjiang Sijing Hospital Shanghai China

**Keywords:** lncRNA CBR3‐AS1, miR‐3163, prognosis, severe pneumonia

## Abstract

**Introduction:**

Severe pneumonia (SP) is a life‐threatening pulmonary infection characterized by high morbidity, frequent complications, and elevated mortality, with rising global incidence in recent years. This study investigated the prognostic value and molecular mechanisms of lncRNA CBR3‐AS1 (CBR3‐AS1) in patients with SP.

**Methods:**

CBR3‐AS1 and miR‐3163 levels were evaluated by real‐time quantitative polymerase chain reaction (RT‐qPCR). The targeting relationship was confirmed by bioinformatics prediction, dual‐luciferase activity assay, and RNA immunoprecipitation (RIP) assay. Cell viability and related indicators were detected by cell counting kit‐8 (CCK‐8), flow cytometry, and enzyme‐linked immunosorbent assay (ELISA).

**Results:**

Serum analysis revealed markedly upregulated CBR3‐AS1 and downregulated miR‐3163 in SP patients versus healthy controls (HC), with bioinformatic and experimental evidence confirming their targeting relationship. In addition, CBR3‐AS1 and miR‐3163 were influential factors affecting death in SP patients and were sensitive predictors of poor patient prognosis. Mechanistically, silencing CBR3‐AS1 promoted the proliferation of MRC‐5 cells after LPS induction, although the miR‐3163 inhibitor partially abolished these changes. Similarly, oxidative stress and inflammatory markers in MRC‐5 cells were also regulated by CBR3‐AS1 and miR‐3163.

**Conclusions:**

CBR3‐AS1 sponge miR‐3163 mediates MRC‐5 cell viability, oxidative stress, and inflammation levels, which ultimately affects the progression of SP. CBR3‐AS1/miR‐3163 axis represents a promising therapeutic target for improving SP patient outcomes.

## Graphical abstract

This study revealed that the elevated lncRNA CBR3‐AS1 may aggravate oxidative stress and inflammatory injury in patients with severe pneumonia by targeting miR‐3163, and the combined detection can be used as an efficient biomarker to evaluate the prognosis of patients.

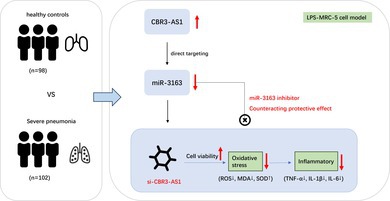

## 1 Introduction

Severe pneumonia (SP) is an acute pulmonary inflammatory disorder characterized by rapid progression and life‐threatening manifestations [1]. Reported mortality rates can be as high as 50%, underscoring its clinical severity [2]. Predominantly affecting immunocompromised populations, including elderly individuals and young children with comorbidities. Patients present clinically with high fever, persistent cough, systemic malaise, and hypotension, often progressing to respiratory failure, multiorgan dysfunction, or septic shock [3]. The persistently high incidence of SP reflects microbial pathogenicity, antibiotic resistance patterns, and prevalent chronic diseases. Current SP management prioritizes anti‐infective therapy alongside respiratory support and organ function maintenance [4]. However, the prevalence of COVID‐19 and the increased drug resistance have complicated the treatment of pneumonia. Consequently, elucidating SP pathogenesis and developing novel therapeutic strategies are essential for optimizing patient outcomes.

Long noncoding RNAs (lncRNAs) function as critical intracellular regulators, containing multiple microRNA (miRNA) binding sites that enable their role as miRNA sponges [5]. LncRNAs are functionally active in various physiological processes with great potential for application. For instance, lncRNA NEAT1 mediates the deterioration of infant pneumonia by targeting miR‐146b [6]. LncRNA HOTTIP functions as a diagnostic indicator for acute respiratory distress syndrome (ARDS) development and patient death, which is implicated in ARDS progression by binding miR‐574‐5p [7]. Additionally, lncRNA DANCR, MEG3, and PVT1 contribute to respiratory disorders via distinct molecular mechanisms [8, 9, 10]. CBR3‐AS1 was initially found to be overexpressed in prostate cancer [11]. Interestingly, CBR3‐AS1 was also reported to be enriched in lung cancer and ulcerative colitis [12, 13]. A recent study elucidated the outstanding role of CBR3‐AS1 in rats with pulmonary fibrosis and its role as a regulator of pulmonary fibrosis by targeting the miRNA‐29/FIZZ1 axis [14]. The predictive performance and molecular function of CBR3‐AS1 in SP patients remain poorly characterized.

This work focuses on the potential of CBR3‐AS1 in SP. We determined the CBR3‐AS1 levels in the serum of randomly screened patients with SP and assessed its relationship with clinical indicators of the patients. Furthermore, the downstream targets of CBR3‐AS1 were predicted by in vitro cellular assays, and the ability of dysregulated CBR3‐AS1 to modulate MRC‐5 cell function, oxidative stress levels, and inflammatory responses was analyzed. In this way, we will reveal the pathological mechanism of CBR3‐AS1 in SP and provide a new interventional target for patient survival.

## 2 Materials and Methods

### 2.1 Recruitment of Subjects

From June 2023 to December 2024, we randomly recruited 200 participants at Shanghai Songjiang Sijing Hospital, including 102 SP cases and 98 healthy controls (HC) from routine health screenings. Eligible SP patients were adults (age > 18 years) with (i) new severe pneumonia [15] and (ii) first‐time diagnosis and treatment at our institution. Exclusions include (i) comorbid cardiovascular or cerebrovascular diseases, (ii) cognitive impairment or psychiatric disorders, and (iii) other major systemic or hereditary diseases.

After receiving standardized clinical therapy, SP patients underwent a 28‐day follow‐up. Follow‐up information was collected via outpatient revisit and telephone interview. Taking death as the primary endpoint, SP patients were further stratified into the survival group and death group based on their survival status on the 28th day.

All experiments were conducted in accordance with the standards of the Ethics Committee of Shanghai Songjiang Sijing Hospital, with all participants providing written informed consent prior to enrollment.

### 2.2 Acquisition of Serum Samples

Demographic and clinical data were recorded for all participants. Fasting peripheral blood samples were collected, processed by centrifugation to isolate serum (3000 r/min for 10 min), and stored at −80°C until batch analysis.

### 2.3 Culture, Treatment, and Transfection of Cell Lines

The human lung fibroblast cell lines MRC‐5 were obtained from the Chinese Academy of Sciences (Shanghai, China), which were maintained in Dulbecco's modified Eagle's medium (DMEM) (Thermo Fisher Scientific, USA) with 10% fetal bovine serum (FBS; Sigma‐Aldrich, USA) at 37°C under 5% CO_2_ in a humidified incubator. The acquisition of the in vitro cell models of pneumonia requires stimulation of MRC‐5 cells with 10 μg/mL lipopolysaccharide (LPS; Sigma‐Aldrich, USA) for 48 h.

Cell transfection assays were performed by referring to the Lipofectamine 2000 reagent manufacturer's guidelines (Invitrogen, USA). Silencing of CBR3‐AS1 (si‐CBR3‐AS1), deregulation of miR‐3163 (miR‐3163 mimic, miR‐3163 inhibitor), and the corresponding negative controls were purchased from RiboBio (Guangzhou, China).

### 2.4 Assessment of RNA Content

Total RNA and the circular DNA (cDNA) synthesis were extracted and performed with Trizol reagent and Revert Aid First cDNA Synthesis Kit (Thermo, USA) according to the manufacturer's instructions, respectively. RT‐qPCR was performed on a StepOne realtime‐PCR system (Applied Biosystems) with a SYBR premix Ex Taq qPCR Kit (Takara, Japan) using glyceraldehyde 3‐phosphate dehydrogenase (GAPDH) and small nuclear RNA (U6) as reference genes. The amount of template was calculated based on the detected Ct value and represented the expression amount of the CBR3‐AS1 and miR‐3163.

### 2.5 Screening and Validation of CBR3‐AS1 Target Genes

LncBook 2.0 public website (https://ngdc.cncb.ac.cn/lncbook/) for the prediction of miRNA with complementary sites to the CBR3‐AS1. The wild‐type plasmid (WT‐CBR3‐AS1) containing the binding sites of CBR3‐AS1 and miR‐3163 as well as the mutant plasmid (MUT‐CBR3‐AS1) with a specific mutation of the binding sites was synthesized based on the pGL3 plasmid (GeneCopoeia, Guangzhou) by RiboBio. MRC‐5 cells were co‐transfected with the plasmid alongside either miR‐3163 mimic or inhibitor, followed by luciferase activity measurement using the Dual‐Luciferase Reporter Assay System (Promega Corporation, USA).

### 2.6 RIP Assay

The interaction between CBR3‐AS1 and miR‐3163‐associated proteins was evaluated using the Magna RIP Kit (Millipore, USA). After treatment of MRC‐5 cells by RIP lysis buffer, MRC‐5 cells were incubated at low temperature overnight after addition of anti‐Ago2 antibody or control anti‐IgG antibody. The protein complexes were digested with proteinase K for 30 min, and the CBR3‐AS1 and miR‐3163 levels were quantified by RT‐qPCR.

### 2.7 Determination of Cell Viability

MRC‐5 cells were transferred to 96‐well plates (2 × 10^3^ cells/well) for 24 h and then supplemented with CCK‐8 reagent (Dojindo, Japan) at specific time points. The incubation was continued for 2 h, and OD was measured at 450 nm.

### 2.8 Determination of Cellular Reactive Oxygen Species (ROS)

MRC‐5 cells were cultured in diluted DCFH‐DA and kept for 20 min. The cells were washed with PBS to remove the DCFH‐DA that did not enter the cells, and then the reactive oxygen species (ROS) levels were assayed by flow cytometry. The ROS assay kit was provided by Solebol (Beijing, China).

### 2.9 Evaluation of Oxidative Stress and Inflammatory Markers

Cellular SOD and MDA levels were quantified using commercial assay kits (Beyotime, China), while inflammatory markers (TNF‐α, IL‐6, and IL‐1β) were measured with ELISA kits (Solarbio, China). Specific procedures were followed as described in the kit instructions.

### 2.10 Statistical Analysis

All data were processed with GraphPad Prism and SPSS software. Differences between two groups were tested by Student's *t*‐test, and multiple groups were performed by one‐way ANOVA with Tukey's post hoc test. The correlation between CBR3‐AS1 and miR‐3163 was performed by Pearson analysis, and the prognosis of SP patients was verified through receiver operating characteristic (ROC) curve. The independent influencing factors of death in SP patients were determined by logistic analysis. The differences in clinical indicators between the survival group and the death group were analyzed by χ^2^ test. *p* < 0.05 was considered statistically significant.

## 3 Results

### 3.1 Clinical Data Analysis of Patients With SP

Table 1 states the baseline data of the included subjects (HC and SP groups). The differences between the two groups in terms of general information (age, BMI, sex, smoking, and drinking) were not statistically significant (*p* > 0.05). However, SP patients exhibited significantly elevated white blood cell count (WBC), C‐reactive protein (CRP), and procalcitonin (PCT) concentration compared to HC group (all *p* < 0.001).

**TABLE 1:** Comparison of baseline characteristics between HC and SP patients.

Indicators	Subjects (*n* = 200)		*p*
	HC (*n* = 98)	SP (*n* = 102)	
Age, years	61.16 ± 4.78	61.85 ± 4.64	0.300
BMI (kg/m^2^)	24.46 ± 2.89	24.89 ± 2.09	0.221
Sex (male/%)	53/54.10	49/48.0	0.393
Smoker (*n*/%)	47/48.0	57/55.9	0.262
Drinker (*n*/%)	45/45.9	53/52.0	0.393
White blood cell count (10^9^/L)	8.33 ± 1.75	10.45 ± 3.54	< 0.001
C‐reactive protein (mg/L)	3.07 ± 1.48	44.40 ± 8.05	< 0.001
Procalcitonin (ng/L)	0.15 ± 0.07	3.15 ± 1.21	< 0.001

*Note:* Data are expressed as mean ± standard deviations.

Abbreviations: BMI, body mass index; HC, healthy controls; SP, Severe pneumonia.

### 3.2 CBR3‐AS1 Was Upregulated in SP

**FIGURE 1:**
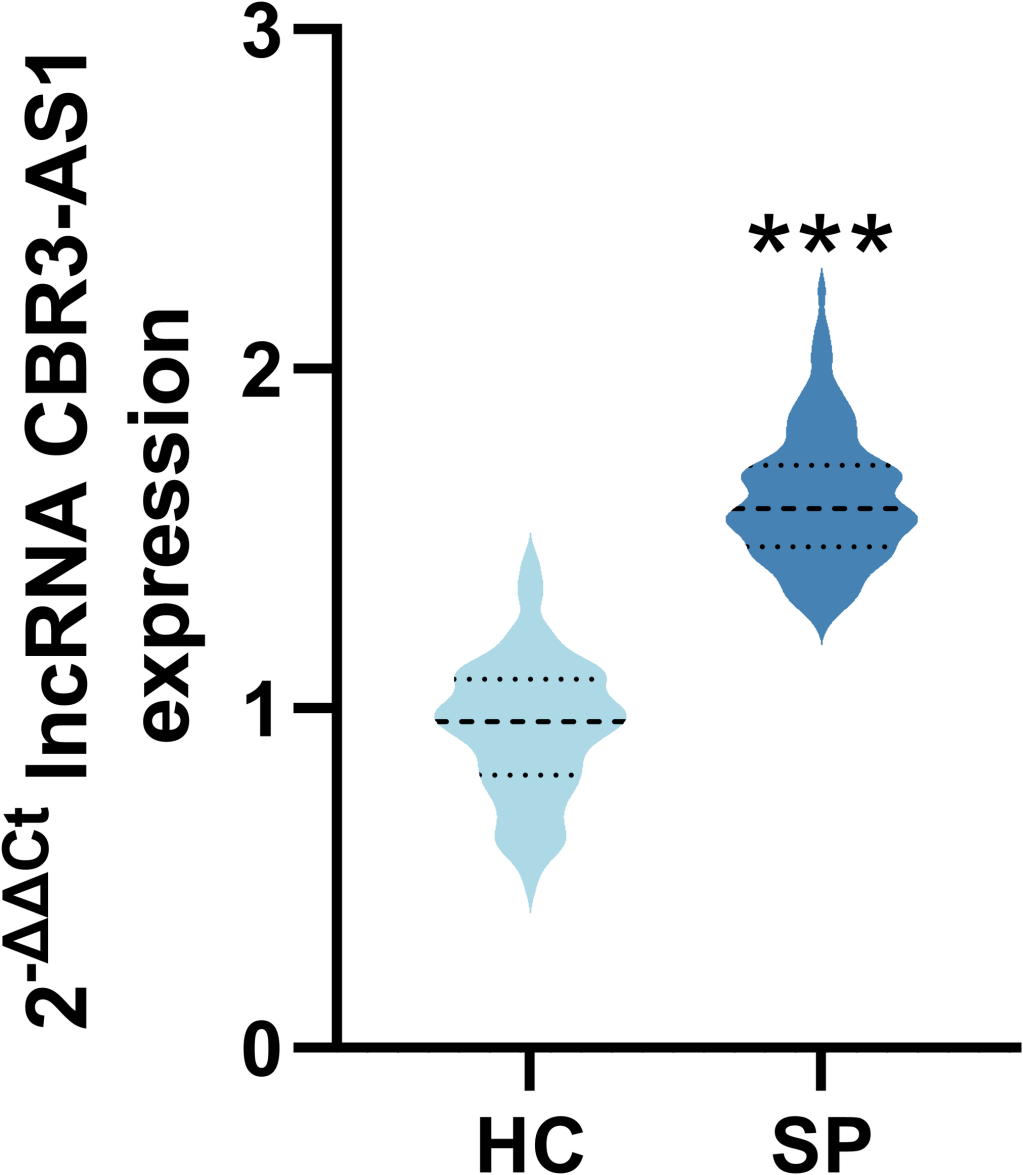
CBR3‐AS1 was upregulated in the SP group (*n* = 102) compared to the HC group (*n* = 98) (***p < 0.001).

Elevated CBR3‐AS1 expression was found in patients with SP compared with healthy individuals as measured by CBR3‐AS1 expression in the serum levels of participants (Figure 1), indicating its potential involvement in SP pathogenesis.

### 3.3 Detection of Downstream Targets of CBR3‐AS1

**FIGURE 2:**
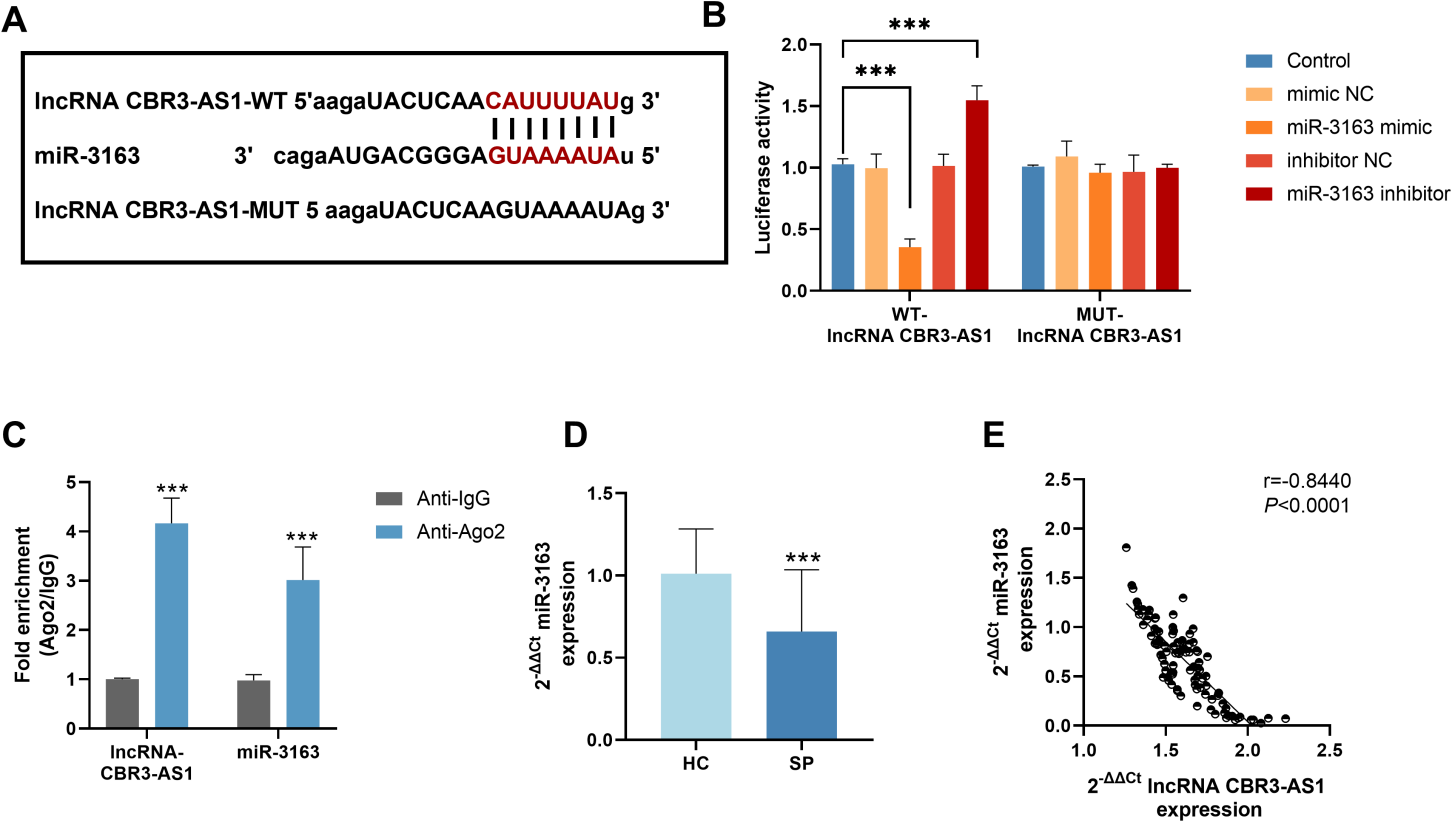
Targeting relationship between CBR3‐AS1 and miR‐3163. (A) Targeted sites with CBR3‐AS1 and miR‐3163. (B,C) The relationship between CBR3‐AS1 and miR‐3163 was further confirmed by luciferase activity and RIP assays (*n* = 3 independent replicates). (D) miR‐3163 was reduced in SP patients (*n* = 102). (E) Correlation between serum CBR3‐AS1 and miR‐3163 expression (*n* = 102; r = −0.8440, *p* < 0.0001) (**p < 0.01, ***p < 0.001).

The target genes of CBR3‐AS1 were predicted by LncBook 2.0 online tool, and complementary binding sites were found (Figure 2A). Furthermore, the dual‐luciferase reporter assay confirmed that co‐transfection of miR‐3163 mimic and WT‐CBR3‐AS1 downregulated the luciferase activity, whereas co‐transfection of miR‐3163 inhibitor and WT‐CBR3‐AS1 increased the luciferase activity (Figure 2B). RIP assay also revealed that CBR3‐AS1 and miR‐3163 were enriched in the anti‐AgO2 group in Figure 2C. miR‐3163 was considered as the direct downstream target of CBR3‐AS1. Quantitative analysis revealed reduced serum miR‐3163 levels in SP patients compared to HC (Figure 2D), which was inversely proportional to CBR3‐AS1 level (Figure 2E; *r* = −0.8440, *p* < 0.0001).

### 3.4 Diagnostic Potential of CBR3‐AS1 and miR‐3163

According to the prognosis results of SP patients, 102 patients were divided into prognostic survival group (*n* = 62) and death group (*n* = 40). Table 2 summarizes the clinical indicators for the different groups of patients. The WBC (*p* = 0.027), CRP (*p* = 0.005), and PCT (*p* < 0.001) in the death group were higher than those of the survival group.

**TABLE 2:** Comparative analysis of indicators among different prognostic groups in SP patients.

Indicators	Prognosis (*n* = 102)		*p*
	Survival (*n* = 62)	Death (*n* = 40)	
Age, years	61.67 ± 4.75	62.13 ± 4.50	0.633
BMI (kg/m^2^)	24.75 ± 1.95	25.11 ± 2.32	0.424
Sex (male/%)	27/43.5	22/55.0	0.258
Smoker (*n*/%)	34/54.8	23/57.5	0.792
Drinker (*n*/%)	31/50.0	22/55.0	0.622
White blood cell count (10^9^/L)	9.83 ± 3.05	11.42 ± 4.04	0.027
C‐reactive protein (mg/L)	42.51 ± 6.86	47.33 ± 8.94	0.005
Procalcitonin (ng/L)	2.71 ± 1.03	3.83 ± 1.17	< 0.001

*Note:* Data are expressed as mean ± standard deviations.

Abbreviations: BMI, body mass index; SP, severe pneumonia.

**FIGURE 3:**
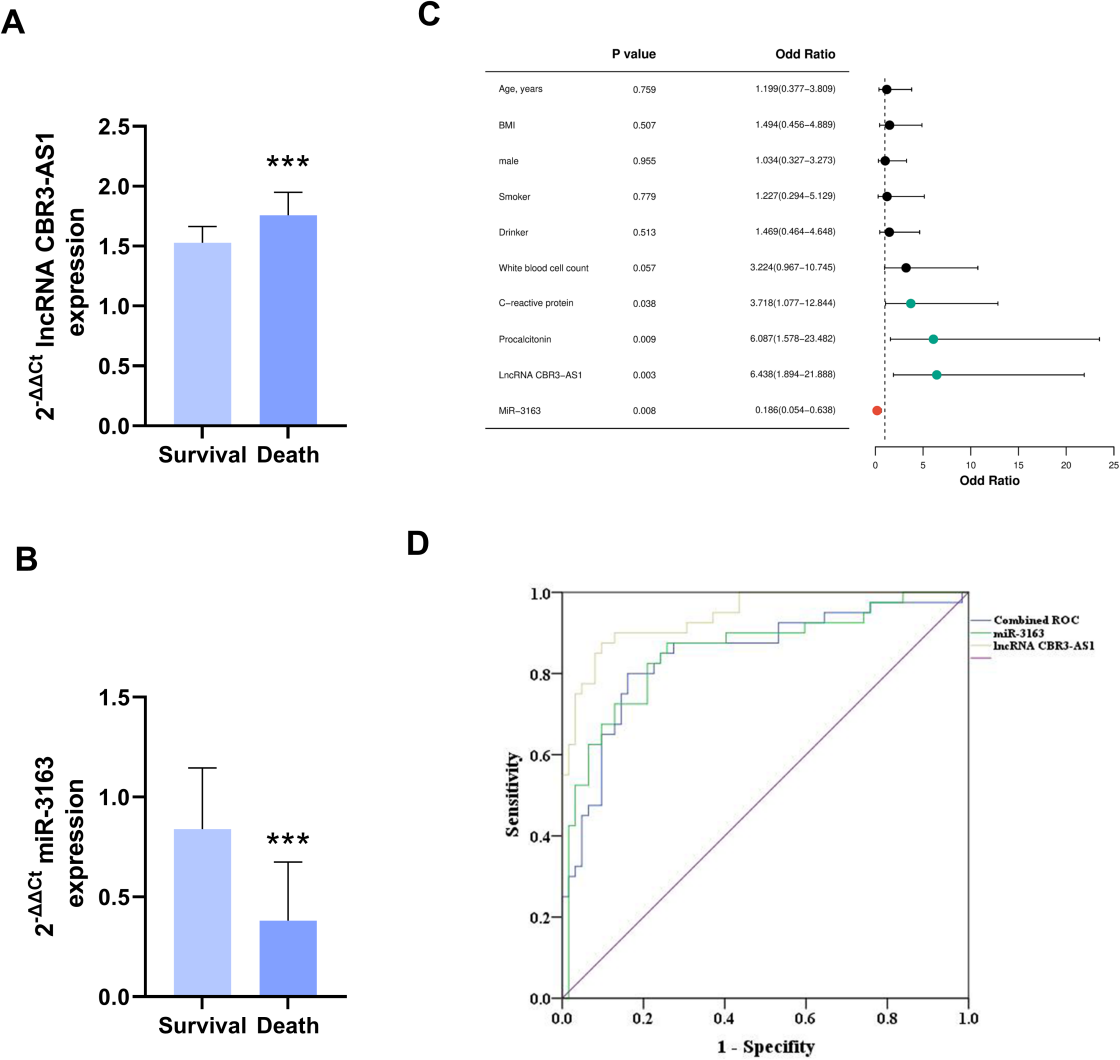
Risk indicators for prognostic outcomes in SP patients. (A,B) CBR3‐AS1 and miR‐3163 levels in survival (*n* = 62) and death groups (*n* = 40) (***p < 0.001). (C) The independent influencing factors of death in SP patients were analyzed by logistic regression (*n* = 102). (D) The ability of CBR3‐AS1 and miR‐3163 to diagnose the prognosis of SP patients through ROC (*n* = 102).

RT‐qPCR assays were performed on the CBR3‐AS1 and miR‐3163 in the serum of patients in two groups. In Figure 3A,B, serum CBR3‐AS1 levels were higher in the death group than the survival group, whereas the miR‐3163 content showed an opposite trend. Moreover, in addition to CRP and PCT, CBR3‐AS1 (*p* = 0.003, odds ratio = 6.438/1.894–21.888) and miR‐3163 (*p* = 0.008, odds ratio = 0.186/0.054–0.638) were also independent risk factors for the death of SP patients (Figure 3C). The ROC curve confirmed that both CBR3‐AS1 and miR‐3163 have predictive potential in the adverse prognosis of SP patients, and the AUC of combined diagnosis of the prognostic outcome of patients was 0.943 (Figure 3D and Table 3).

**TABLE 3:** Diagnostic efficacy of lncRNACBR3‐AS1 and miR‐3163 in predicting prognosis differences in SP patients.

Items	LncRNA CBR3‐AS1	miR‐3163	LncRNA CBR3‐AS1 combined with miR‐3163
AUC	0.847	0.855	0.943
Sensitivity (%)	80.0	87.5	87.5
Specificity (%)	83.9	74.2	90.3
95% CI	0.765–0.929	0.775–0.935	0.900–0.86
*p*	< 0.001	< 0.001	< 0.001

Abbreviations: AUC, area under the curve; SP, severe pneumonia.

### 3.5 Effect of CBR3‐AS1 and miR‐3163 Knockdown on Cell Function

**FIGURE 4:**
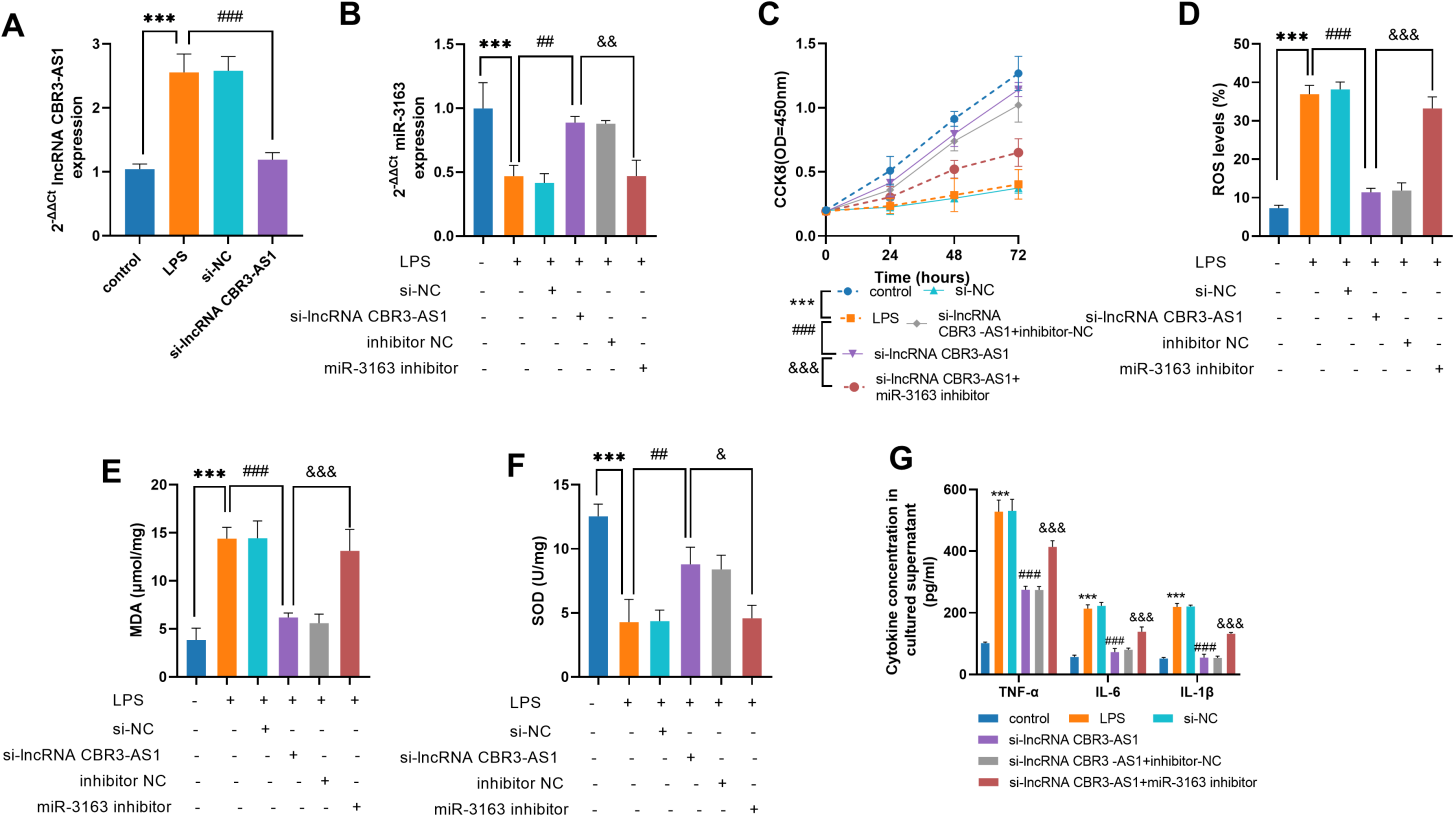
Downregulation of CBR3‐AS1 and miR‐3163 mediated cell viability, oxidative stress, and inflammatory response. (A) Dysregulated transfection efficiency of CBR3‐AS1 (*n* = 3 independent replicates; ***p < 0.001 vs. control; ^###^ *p* < 0.001 vs. LPS). Regulation of (B) miR‐3163 expression, (C) cell viability, (D–F) oxidative stress levels, and (G) inflammatory indicators by LPS induction and cotransfection of si‐CBR3‐AS1 and miR‐3163 inhibitor (*n* = 3 independent replicates; ***p < 0.001 vs. control; ^##^ *p* < 0.01, ^###^ *p* < 0.001 vs. LPS; ^&^ *p* < 0.05, ^&&^ *p* < 0.01, ^&&&^ *p* < 0.001 vs. si‐CBR3‐AS1).

LPS‐induced MRC‐5 cells elucidated enhanced CBR3‐AS1 expression, which was attenuated by si‐CBR3‐AS1 transfection (Figure 4A). LPS treatment downregulated miR‐3163 in MRC‐5 cells, whereas CBR3‐AS1 silencing reversed this suppression. Subsequent miR‐3163 inhibitor restored baseline expression levels (Figure 4B). After LPS intervention, the proliferation ability of MRC‐5 cells was decreased. When the CBR3‐AS1 level was suppressed, cell growth ability was upregulated, whereas co‐transfection with miR‐3163 inhibitor partially offset the positive effects of CBR3‐AS1 (Figure 4C).

As suggested by the detection of oxidative stress indicators and inflammatory factor concentrations in the cells, LPS treatment of MRC‐5 cells resulted in increased ROS and MDA levels and decreased SOD levels, whereas the inhibition of CBR3‐AS1 expression attenuated LPS‐induced effects. Alternatively, this attenuating effect was reversed after miR‐3163 inhibitor transfection (Figure 4D–F). TNF‐α, IL‐1β, and IL‐6 levels were upregulated by LPS induction, and CBR3‐AS1 silencing ameliorated the inflammatory response, which was partially abolished by the miR‐3163 inhibitor (Figure 4G).

## 4 Discussion

The pathogenesis of SP involves intricate inflammatory dysregulation and immune dysfunction, culminating in significant pulmonary tissue injury and respiratory impairment [16, 17]. To minimize the threat of SP to patient survival, more sensitive biomarkers are needed to provide relevant diagnostics and predictions to inform clinical treatment.

Accumulating evidence states that lncRNAs modulate target miRNAs expression across pneumonia progression stages and cellular developmental processes [18]. In a study by Gao et al., SNHG16 was weakened in pneumonia and accelerated LPS‐induced apoptosis via mediating miR‐210 [19]. Cao et al. proposed that LncRNA RP11‐773H22.4 is positively expressed in SP and has diagnostic and prognostic value in patients. RP11‐773H22.4/miR‐1287‐5p axis affects the viability and inflammatory expression of MRC‐5 cells [20]. Here, we verified that CBR3‐AS1 is upregulated in the serum of SP patients and miR‐3163 is its downstream target, which is downregulated in SP. Meanwhile, CBR3‐AS1 was quantitatively overexpressed in ulcerative colitis, breast cancer, and lung cancer [21, 22, 23]. miR‐3163 also revealed to be decreased expression in lung cancer in the available literature [24], which is consistent with our findings. In tuberculosis, miR‐3163 was also identified as downregulated and functionally contributes to disease progression [25]. The findings indicate that CBR3‐AS1 appears to drive SP pathogenesis via miR‐3163 inhibition, as mechanistically demonstrated. Moreover, SP patients were stratified into survival and death groups based on 28‐day clinical outcomes. Elevated CBR3‐AS1 expression coupled with reduced miR‐3163 levels correlates with adverse clinical outcomes in SP patients, and their combined assessment improves prognostic accuracy. The above implies that both CBR3‐AS1 and miR‐3163 may act as prognostic biomarkers in SP.

Lung fibroblasts are activated after the onset of pneumonia and promote lung fibrosis [26]. LPS treatment of MRC‐5 cells induced the release of pro‐inflammatory factors and cell transformation, which was used to construct the model of pneumonia cells and was suitable for studying the mechanism of SP [27]. In this study, we induced MRC‐5 cells using LPS and successfully regulated the CBR3‐AS1 and miR‐3163 levels in the cells by transfecting them with silencing CBR3‐AS1 and miR‐3163. LPS treatment significantly inhibited MRC‐5 cell proliferation, concurrently elevating oxidative stress markers and pro‐inflammatory cytokine secretion. The induction of MRC‐5 cells by LPS stimulates cellular inflammatory responses; oxidative stress and apoptosis were also mentioned in Zhuang et al. [28]. This is consistent with our experimental results. Oxidative stress markers and inflammatory mediators are critically involved in the pathogenesis of pneumonia [29]. Among them, ROS levels directly indicate intracellular oxidative stress intensity. MDA, as the end product of lipid peroxidation, quantifies cell membrane oxidative damage, and SOD activity serves as a biomarker of cellular antioxidant capacity [30]. Knockdown of CBR3‐AS1 markedly reduced ROS and MDA content but promoted SOD activity. This change was reversed by co‐transfection of miR‐3163 inhibitor. Similarly, downregulation of CBR3‐AS1 attenuated the expression of inflammatory markers in MRC‐5 cells after LPS induction, whereas the introduction of miR‐3163 partially reversed the function of CBR3‐AS1. Our study elucidated that inhibition of the CBR3‐AS1/miR‐3163 axis may alleviate the progression of SP by suppressing oxidative stress and inflammatory.

However, the LPS‐induced MRC‐5 cell model may not fully mimic the complex pathophysiological process of SP. The occurrence of SP in vivo involves a multifaceted immune microenvironment and the interaction of multiple cell types. Therefore, our in vitro findings need to be further validated by in vivo models. Additionally, to further improve the rigor and statistical validity of our results, further accumulation of samples in clinical work is still needed in the future. The downstream targets of the CBR3‐AS1 and miR‐3163 axis should be explored more deeply.

In summary, CBR3‐AS1 was enhanced in the serum of SP patients, whereas miR‐3163 was negatively expressed. Both serve as independent risk factors, and their combination yields better diagnostic value for predicting poor prognosis. Mechanistically, downregulation of CBR3‐AS1 sponges miR‐3163 affected cell activity and alleviated stress and inflammation levels. This finding provides a new perspective for the treatment and survival prediction of SP patients.

## Author Contributions

C.L., M.L., J.Y. and P.L. jointly reviewed the literature and carried out the experimental design. C.L., M.L., J.Y. and P.L. performed data analysis, J.Y. wrote the manuscript and performed article integration. All authors agree to publish this study for publication.

## Funding

The authors have nothing to report.

## Ethics Statement

The study was performed in line with the principles of the Declaration of Helsinki. Approval was granted by the Ethics Committee of Shanghai Songjiang Sijing Hospital before the study began. The participants' right to be informed about the study was ensured and agreed to participate in the study.

## Conflicts of Interest

The authors declare no conflicts of interest.

## Data Availability

The data that support the findings of this study are available from the corresponding author upon reasonable request.
